# The Associations among Dental Anxiety, Self-Esteem, and Oral Health-Related Quality of Life in Children: A Cross-Sectional Study

**DOI:** 10.3390/dj11070179

**Published:** 2023-07-21

**Authors:** Amjad Alharbi, Gerry Humphris, Ruth Freeman

**Affiliations:** 1 DHSRU, Dundee Dental School and Hospital, University of Dundee, Dundee DD1 4HN, UK; 2 College of Dentistry, Qassim University, Buraydah 51452, Saudi Arabia; 3 Health Psychology, School of Medicine, University of St Andrews, Edinburgh KY16 9AJ, UK; 4 Public Health, NHS Tayside, Dundee DD2 1UB, UK

**Keywords:** dental anxiety, self-esteem, child oral health-related quality of life, child, adolescents

## Abstract

Background: The current evidence on the relationships among child oral health-related quality of life, dental anxiety, and self-esteem indicates that we need to investigate these relationships to improve our understanding of the associations. Therefore, the current research aimed to enhance this evidence and provide an overview of the participating children’s oral-health-related quality of life (as measured by the CPQ_8–10_), self-esteem (as measured by the Coopersmith SEI-SF), and dental anxiety (as measured by the CFSS_DS) and how these child-related outcome measures interacted and were related to one another. Method: A cross-sectional survey was conducted on a random sample of school children (*n* = 1900) aged 8 to 10 years. The questionnaire was collected through validated self-report measures: dental anxiety, COHRQoL, and self-esteem. Structural equation modelling (SEM) was used to test the strength of the association of our model to explore the relationships among these three psychological constructs. The moderating effects of age, gender, location, and the educational board were analysed for their possible influence on these relationships. Results: Significant relationships between COHRQoL and child dental anxiety and between COHRQoL and SE were detected. The relationship subscale between COHRQoL and child dental anxiety was 0.24, (*p* < 0.001). A stronger correlation between COHRQoL, and SE was found, with B = −0.77, (*p* < 0.001). Although the association between CDA and SE was small, it was statistically significant (*p* = 0.03). These findings provide some important background information for designing effective educational programs for children.

## 1. Introduction

Dental anxiety can lead to the avoidance of dental treatment [1], which, over time, may lead to poorer oral health [2], and together with health-related and psychosocial outcomes [3], may increase dissatisfaction with facial and dental appearance [4]. Lower satisfaction with the appearance of teeth may be associated with embarrassment and feeling tense [5], fostering low self-esteem. Children experiencing high levels of dental anxiety have been found to have poorer oral health outcomes, such as a greater prevalence of dental caries [6], and they experience more pain and discomfort [7] than non-dentally anxious children [8]. In addition, poorer oral health has been recognised to have negative influences on children’s ability to socialise with others and complete their schoolwork, thereby potentially affecting their quality of life. Consequently, it may be proposed that dental anxiety could negatively impair children’s self-esteem and their oral health-related quality of life [9]. Furthermore, self-esteem was also found to have a negative influence on COHRQoL. Aguo et al. reported that self-esteem is a determinant of COHRQoL in children seeking orthodontic treatment [10]. A longitudinal study found that there were significant longitudinal associations between the changes in COHRQoL and changes in self-esteem in those seeking orthodontic treatment [11]. Hence, we propose to investigate the underlying profile of a number of key dental-related constructs held by children, including their dental anxiety, their beliefs about the impact of oral health, and their sense of identity. This more inclusive approach to understanding children may be predictive of their response towards new educational oral health programmes. Therefore, we believe it is important to invest substantial effort in constructing a wider working model to describe children’s views about oral health based on our previous systematic review [12].

Previous work has shown that, with this age group, the specific benefits of a conventional, and perhaps limited, dental health education programme warrants a more comprehensive approach. A systematic review found that a moderate relationship exists between a child’s oral health-related quality of life (COHRQoL) and their self-esteem (SE) and a small correlation between a child’s oral health-related quality of life and child dental anxiety (CDA). However, very little evidence could be found from the systematic review to support a connection between child dental anxiety and self-esteem [13]. In this review, Alharbi and colleagues suggest that a new investigation is needed, with good recruitment, to examine closely the inter-relationships of these three psychological constructs using a detailed statistical approach. The majority of the research in this field has been reported in the US, Europe, and Australia. To broaden our focus to another region of the world appears timely with global steps to improve health education [14]. Therefore, this study aimed to explore, in Saudi Arabia, the relationships among these three psychological constructs, that is, COHRQoL, SE, and CDA, using cross-sectional study data and to examine the effects of gender, age, location, and the educational board on the model framework. It was hypothesised that there are significant relationships between COHRQoL and CDA, COHRQoL and SE, and CDA and SE.

## 2. Materials and Methods

### 2.1. Sampling

Samples of children were collected from November 2019 to January 2020 from Qassim Province, Saudi Arabia. There was a total of 95,100 students attending public primary school under two different educational boards. Schools in urban and semi-urban areas in Qassim Province were selected randomly by a computer algorithm using a random number generator. The sample size was calculated based on a power calculation to achieve 80% power for a small effect with a significance level (alpha) of 0.05 using a two-sided paired *t*-test. With a 20% loss of follow-up, the total sample size required was 1688 children. The inclusion criteria for the schools were having the minimum required sample size of 50 students. All children who attended the sampled schools were aged between 8 and 10 years of age, who assented, and whose parents provided written consent were invited to participate. The exclusion criteria were schools with samples smaller than 50 and who refused to participate.

### 2.2. Measures

The questionnaire was collected through a set of validated self-report measures. The Child Perception Questionnaire (CPQ_8–10_) was used to assess the COHRQoL. The CPQ_8–10_ is a self-report questionnaire developed by Jokovic et al. [15]. The questionnaire asks children about the frequency of oral health impacts and if they had experienced any of these impacts, e.g., painful teeth, difficulty speaking or eating in the previous four weeks. The CPQ_8–10_ consists of twenty-five items to measure the COHRQoL using a five-point Likert scale, with higher scores indicating greater oral health impacts and poorer COHRQoL. The English version of the CPQ_8–10_ was found to be a reliable and valid scale [16]. The Arabic version of the CPQ_8–10_ scale’s consistency and reliability were found to be high, with a Cronbach α of 0.90 [15].

The Coopersmith Self-Esteem Inventory-School Form (Coopersmith SEI-SF) is a self-report questionnaire and is used to measure self-esteem in 8–15-year-old children. A short form was developed by Coopersmith in 1981 to be used when time is limited [14] and was used here to assess children’s SE. The children are asked to answer a set of eight questions and whether they agree (“like me”) or do not agree (“not like me”) with the statement. Scores range from 8 (high self-esteem) to 0 (low self-esteem). The English version of the SEI-SF is a reliable and valid scale [17]. Similarly, the Coopersmith SEI-SF scale was translated initially from English to Arabic. The internal consistency and reliability were found to be acceptable and have a Cronbach α of 0.60 [15,16,17,18].

The CFSS-DS, developed in 1982, is a well-known instrument for evaluating dental anxiety in children [19]. It is a self-administered questionnaire for children aged 5 to 15 years old. The child is asked ‘How afraid are you of? …’, and then responds to 15 items related to different aspects of the dental situation, such as fear of the dentist, dental treatment, injection, and choking. Their responses are assessed on a 5-point Likert scale, with scores of 1 (not afraid), 2 (slightly afraid), 3 (fairly afraid), 4 (quite afraid), and 5 (very afraid), and giving total scores ranging from 15 to 75, with a higher score indicating dental fear. The Arabic version of the CFSS-DS was found to be a reliable and valid measure of dental anxiety in Arabic-speaking children (Cronbach α = 0.78) [20].

Although the questionnaire might be long and time-consuming for children, the study aimed to include children as respondents rather than proxies (e.g., parents).

Bell et al. recommended that when designing a questionnaire, the decisions to use a questionnaire for children should be based on the content of the questionnaire rather than the length of the questionnaire. The questions should be appropriate and simple, with straightforward syntax for children. Therefore, with careful consideration of the content, and the essential variables required to be assessed, researchers are encouraged to continue using questionnaires in a school setting but only to collect what is strictly necessary [21].

Upon collection of the questionnaires from all schools, the responses were coded and entered in digital format for checking and data cleaning.

### 2.3. Statistical Analysis

The scales were checked for factorial consistency using both exploratory and confirmatory factor analytical procedures [15] and compared with previous research using identical instruments. Descriptive analyses were conducted to provide conventional summary statistics of the means and standard deviations for the continuous variables and percentages for the categorical variables. The statistical analysis was conducted using STATA software (Release 16, Stata Corp LLC, College Station, TX, USA) [22]. Student’s *t*-tests, multiple comparison tests, and chi-square analyses were run for basic descriptions and understanding of the variation across the contextual variables, namely age, sex, location, and educational board. Scattergrams of pairwise associations were inspected for outliers and corrected where necessary with reference to the original questionnaire copies. An alpha level of 0.05 (two-sided) was used throughout.

Structural equation modelling (SEM) was used to build a model that consisted of the latent variables of dental anxiety, COHRQoL, and child self-esteem. The model enabled simultaneous testing at the same phase of completion of the questionnaire. Each latent variable was defined by a set of ‘indicator’ variables, that is, the raw responses from the child’s questionnaire. The advantage of this methodology was the ability to use these latent variables to assess the key hypothesised relationships that crucially were disattenuated from the measurement error. In other words, the bias created when using raw variables, which inevitably include rating errors, was diminished. The analyses were run using discrete ‘parcels’ of items rather than individual items [23]. The parcels were determined from the confirmatory factor analyses. Each parcel was indicated by how well items within each of the scales clustered together. The advantage of using parcels in the SEM procedures was that the extreme estimation task attempted by the statistical software of using individual items (over 30+ items) was avoided. Further, the small number of ‘indicator’ variables that defined the latent variables ranged from two to three, aiding the interpretation and manageability of the computational task. All of the indicator variables were aggregations of items that were typically summed totals of each of the factors known to exist from the first stage of analysis, and the distributional properties of the data were conducive to the assumptions required of the maximum likelihood estimation, e.g., not heavily skewed or suffering substantial kurtosis.

### 2.4. Procedure

The investigator in the field (AA) attended each classroom for 45 min at every school selected, accompanied by the classroom teacher. The latter helped to answer questions from the children during the administration of the questionnaire, maintaining a supportive environment for the children to concentrate and providing encouragement. Children experiencing trouble with responding were counselled without influencing their responses.

## 3. Results

A total of 1900 children aged 8 to 10 years old were included in the study. Sixty-two cases with missing values were excluded from the analysis. All of the schools that took part were public (non-fee-paying) schools. The schools were divided by location; 13 schools were from semi-urban areas, and 20 schools were from urban areas. Table 1 shows the proportions of children residing in urban and semi-urban localities, their gender, and age groups in years.

To investigate the associations among COHRQoL (CPQ_8–10_), SE (SEI-SF), and CDA (CFSS-DS), a covariance (unstandardised) matrix was constructed to examine all the sub-scales of the three constructs (i.e., three sub-scales); (i) the dental fear, (ii) the hospital fear, and (iii) stranger fear sub-scales (Supplementary File).

COHRQoL, as measured by the CPQ_8–10_, was shown to have two factors (i.e., two sub-scales): (i) an oral and functional factor and (ii) a psychosocial factor. The mean score for the total CPQ_8–10_ for the whole sample was 36.89 (SD 12.99). Self-esteem, as measured by the SEI-SF, had a factor structure consisting of two factors (i.e., two sub-scales): (i) hesitant and (ii) confidence sub-scales, with a mean score of 6.29 (SD 1.8). As previously mentioned in the methods for the CFSS-DS, this was measured already demonstrating a clearly consistent three-factor structure, and the mean for child dental anxiety was 33.31 with (SD 13.71).

The model constructed was based on the hypothesis proposed in the systematic review [13], with seven sub-scales representing COHRQoL (CPQ_8–10_), SE (SEI-SF), and CDA (CFSS-DS) included in the structural equation model (SEM). The standardised model coefficients, *p*-values, and 95% Cis are shown in Table 2. The factor loadings were all highly statistically significant (*p* < 0.001) and above 0.40, except for the SE2 (confidence) sub-scale, where the loading was 0.20.

**Figure 1:**
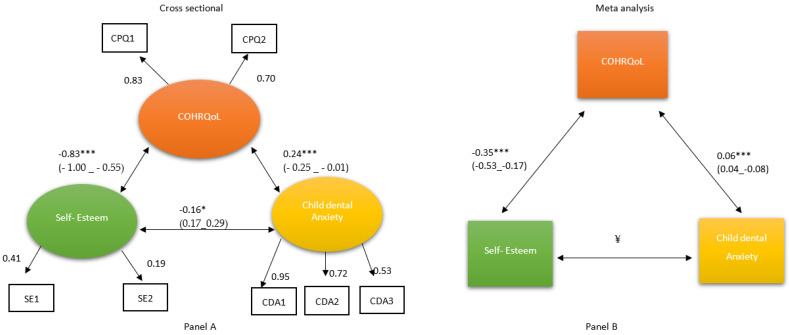
Comparison between Panel A, the structural equation model (standardised solution), and Panel B, the meta-analysis model from the systematic review [10]. CPQ 1 and 2: Child Perception Questionnaire, oral and functional factor (CPQ 1) and psychosocial (CPQ 2) subscales. SE 1 and 2: Self-esteem hesitant (SE 1) and confidence (SE 2) subscales. CDA 1, 2, 3, CFSS-DS dental fear (CDA 1), hospital fear (CDA 2), and stranger fear (CDA 3) subscales; All coefficients controlled for effects of age, gender, location, and educational board. * *p* < 0.05, ** *p* < 0.01, *** *p* < 0.001; ¥ no appropriate association available from the literature, hence blank; 95% CIs in brackets.

The structural equation model with standardised path loadings (Figure 1, Panel A) showed significant relationships between the COHRQoL and child dental anxiety subscales, with the value of B = 0.24, *p* < 0.001 (note: the standardised values are equivalent to correlations). This indicates that higher levels of dental anxiety were associated with poorer COHRQoL. The path between the latent variables COHRQoL and SE was (B = −0.77, *p* < 0.001) confirming the association between COHRQoL and SE, specifically, that high self-esteem is associated with better COHRQoL. The path association between CDA and SE was statistically significant (B = −0.13, *p* = 0.03) and supported the systematic review hypothetical model.

The SEM model under conventional maximum likelihood estimation required fewer than 10 iterations to converge and returned no Heywood cases (that is, negative measurement errors). The goodness of fit was assessed using several indicators, including the chi-square, and indicated, at first, an unacceptable model fit (chi-square = 117.25, df = 11, *p* < 0.001). The chi-square value was statistically significant, which might have been due to the oversensitivity from possessing a relatively large sample size. Upon further examination, an acceptable model fit should also have an RMSEA of less than 0.08 [24]. According to these criteria, the RMSEA indicator did indeed demonstrate a favourable model fit (RMSEA = 0.07), together with the CFI (0.92) and SRMR (0.036), which also indicate a ‘just acceptable’ model fit [25].

The hypothetical model proposed in the systematic review [13], indicating that there are relationships among DA, SE, and COHRQoL, was confirmed using SEM. Overall, associations were found among the three constructs; the child oral health-related quality of life, self-esteem, and child dental anxiety subscales supported the hypothetical model already presented [13]. To determine if these relationships were similar following the control of contextual variables, a sensitivity analysis was performed. The variables were included as covariates in the model and the analysis was rerun. Table 3 shows the standardised model coefficients, *p*-values, and 95% CIs. All item loadings were significant at the *p* < 0.001 level.

Gender was associated with COHRQoL and SE, and girls’ oral health had a higher impact than that of the boys, while SE was higher in boys. Similarly, location was associated with COHRQoL and SE. The age of the children had a negative correlation with COHRQoL and a positive correlation with SE. Although these associations were statistically significant, they all indicate relatively weak associations (Table 4).

## 4. Discussion

We hypothesised in this investigation that the associations among COHRQoL (CPQ_8–10_), SE (SEI-SF) and CDA (CFSS-DS) would be reliable and show some consistency with previously published work, where available. Significant relationships were demonstrated between COHRQoL and CDA and between COHRQoL and SE, confirming the original meta-analysis set of findings [15]. Figure 1 shows a comparison between the meta-analysis association and the current study’s coefficients. The SEM model shows a set of stronger correlations among all three constructs (COHRQoL and CDA, COHRQoL and SE, and CDA and SE) than the meta-analysis.

In addition, the moderating effects of a number of important demographic data (age and gender) and socially relevant contextual factors (location and educational board) were analysed for their possible influence on these relationships among the three psychological constructs.

Gender, age, location, and educational board were found to have weak effects on the associations among COHRQoL (CPQ_8–10_), SE (SEI-SF), and CDA (CFSS-DS). Gender, age, and location were found to have an impact on COHRQoL and SE. Location had a significant effect on CDA only. Gender was associated with COHRQoL and SE. Girls reported a higher impact of COHRQoL than boys. Also, SE was higher in boys than girls. This is consistent with previous studies finding that girls reported a poorer OHRQoL and lower self-esteem in comparison to boys [26,27]. Moreover, a further study found that boys tended to report higher self-esteem than girls [28]. This might be explained by girls’ tendencies to report lower self-esteem and self-perception of oral health and body image in comparison to boys [29].

The ages of the children were found to influence COHRQoL and SE. Similar findings have been reported, showing that age influences the CPQ overall score [30]. However, one study found that the CPQ scores improved over time regardless of treatment status [31].

The relationships among these three psychological constructs, which are child oral health-related quality of life, self-esteem, and child dental anxiety, have not been studied previously in such an intensive manner. The associations demonstrated in the systematic review and meta-analysis show a moderate relationship between COHRQoL and SE and a small correlation between COHRQoL and CDA [13]. However, there was little evidence from the systematic review to support an association between child dental anxiety and self-esteem [13].

Similarly, we found stronger correlations among the three constructs, specifically between COHRQoL and CDA and between COHRQoL and SE, compared to the meta-analysis.

Although CDA was significantly correlated to SE, the correlation was weak (−0.13). A recent study also found dental anxiety was associated negatively with self-esteem. However, this significant association was significant only for the 17–18 years age group [32]. Three different age groups participated in that particular study. The children and adolescents were aged 11–14 years, 15–16 years, and 16–18 years. In their results, they found that a high level of dental anxiety was not associated with low self-esteem in the lower age group [33]. Age acted as a moderator. This tentative negative relationship may be explained because SE became more salient with children in older age groups, enabling a correlation with dental anxiety to be revealed. That is, only when the age increased did the relationship between dental anxiety and SE become apparent.

An important finding we found from our investigation in Saudi Arabia was that children reported a higher impact of COHRQoL associated with higher CDA scores. This was consistent with other studies where CDA was a significant predictor of impaired OHRQoL [34,35,36,37]. This result may be explained by the process of developing dental anxiety over a longer term, often referred to as a vicious cycle of dental anxiety [2].

In this vicious cycle, dental anxiety leads to avoidance of going to the dentist, which may lead to delayed dental treatment, which in turn results in poor oral health and increased social impact of oral ill-health and, in addition, worse self-rated oral health [2]. A study that analysed the data of 4916 children aged 5 years and 8 years found that higher levels of dental anxiety predicted poorer oral health, and high dental anxiety predicted that the child’s oral health had a greater effect on the quality of family life [38].

Another important finding from this research was that COHRQoL was strongly correlated to self-esteem, showing an inverse relationship, namely that the high impact of quality of life was associated with low self-esteem.

Jokovic et al. concluded that the understanding of complex concepts, such as health and well-being, is influenced by variables such as gender, age, and age-related experiences [36]. A study found that SE may have a possible mediator effect on OHRQoL in children seeking orthodontic treatment. Children with high SE were more likely to report better OHRQoL. This may be because the social and emotional impacts of malocclusion may be influenced by the psychological profile of the child [10].

There were some limitations to this study. First, the study was cross-sectional in nature and did not attempt to determine the causal order. We attempted to construct a model that enabled, if imperfectly, a comparison to what we have reported previously in our structured review, i.e., meta-analysis. It may be possible to report these networks of relationships in the short term, but it may well be that their long-lasting effect is not strong. A rare example of an investigation researching the reverse process, that is, the effect of treatment on psychological makeup, is the Cardiff longitudinal dental study, which followed young adolescents for 20 years and found that orthodontic treatment had little positive impact on psychological health or quality of life in adulthood [39].

The second limitation was that most participants were girls. The boys were a much smaller group. The boys were likely underrepresented in this study due to the education system in Saudi Arabia and not being able to obtain access to boys’ schools. Additionally, although COVID-19 did not affect the sample size directly, the pandemic reduced the sample size included in this study because of the sudden change in education methods from regular face-to-face to online delivery. Nevertheless, the Coopersmith SEI-SF was problematic and did not perform well with Saudi children. A possible explanation for this finding could be that the Coopersmith SEI-SF scale was derived from a scale with a much larger number of items, and therefore, when a small number of items was selected, it may have changed the context, meaning, and properties of the items from the child’s perspective. These findings agree with those of Hills et al. [40]. They concluded that although the scale’s reliability was adequate, the individual item rest-of-test correlations for a couple of the items were relatively small [40].

The main strength of this research was the large sample size of almost two thousand children. A second strength was adopting a structural equation modelling (SEM) approach, which, firstly, permitted a rigorous inspection of ‘correlated residual errors of the individual items and unique loadings to representative constructs’ and, secondly, provide additional evidence for the relationships among the three child-related outcome measures i.e., COHRQoL, SE, and CDA. In addition to the practice of using latent variables in analyses to understand relationships, this approach helped tease out the complexity of the model on how different variables could affect the COHRQoL, SE, and CDA correlations simultaneously and by removing the bias inherent with the inevitable measurement error of rating systems.

## 5. Conclusions

This study aimed to investigate the associations among COHRQoL (CPQ_8–10_), SE (SEI-SF), and CDA (CFSS-DS). Significant relationships were demonstrated among the three constructs, as predicted from a previous structured review, albeit with stronger relationships.

This study was an important step in the process of developing a system of assessment of the psychological constructs. The utilisation of psychological outcome measures will allow for a subjective evaluation of the effect of oral health education interventions on children’s oral health. The research findings suggest that a better understanding of the associations among the three psychological constructs, that is, child oral health-related quality of life, self-esteem, and child dental anxiety, may help to plan appropriate oral health interventions and provide better treatment strategies.

## Figures and Tables

**Table 1:** Distribution of children by location, gender, and age.

	Frequency	Percent
Location of school		
Urban	1076	56.6%
Semi-urban	824	43.3%
Educational Board		
Qassim: Rass	1178	62%
Qassim: Buridah	722	38%
Gender		
Girls	1706	89.7%
Boys	194	10.2%
Age		
8 years	511	26.9%
9 years	547	28.8%
10 years	848	44.3%

**Table 2:** Standardised factor loadings, *p* values, and 95% confidence intervals for COHRQoL, SE, and CDA subscales.

Standardised	Factor Loadings	*p*	(95% CI)
CPQ 1 ¥	0.84	<0.001	0.77–0.90
CPQ 2	0.70	<0.001	0.65–0.76
SE1 ∞	0.41	<0.001	0.28–0.54
SE2	0.20	<0.001	0.12–0.27
CDA 1 ⁂	0.94	<0.001	0.91–0.98
CDA 2	0.72	<0.001	0.69–0.76
CDA 3	0.52	<0.001	0.49–0.56

¥ CPQ 1 and 2: Child Perception Questionnaire oral and functional factor (CPQ 1) and psychosocial (CPQ 2) subscales. ∞ SE 1 and 2: Self-esteem hesitant (SE 1) and confidence (SE 2) subscales. ⁂ CDA 1, 2, 3: CFSS-DS dental fear (CD 1), hospital fear (CDA 2), and stranger fear (CDA 3) subscales.

**Table 3:** Standardised factor loadings, *p* values, and 95% confidence intervals for COHRQoL, SE, and CDA subscales.

Standardised	Factor Loadings	*p*	(95% CI)
CPQ 1 ¥	0.83	<0.001	0.77–0.89
CPQ 2	0.70	<0.001	0.64–0.75
SE1 ∞	0.41	<0.001	0.28–0.52
SE2	0.20	<0.001	0.12–0.26
CDA1 ⁂	0.94	<0.001	0.90–0.97
CDA2	0.72	<0.001	0.68–0.75
CDA 3	0.52	<0.001	0.48–0.56

¥ CPQ 1 and 2: Child Perception Questionnaire oral and functional factor (CPQ 1) and psychosocial (CPQ 2) subscales. ∞ SE 1 and 2: Self-esteem hesitant (SE 1) and confidence (SE 2) subscales. ⁂ CDA 1, 2, 3: CFSS-DS dental fear (CD 1), hospital fear (CDA 2), and stranger fear (CDA 3) subscales.

**Table 4:** Correlations, *p* values, and 95% confidence intervals for COHRQoL, SE, and CDA.

	CPQ	SE	CDA
Gender	−0.12 ***	0.24 ***	−0.03
Age	0.06 *	0.27 ***	0.002
Location	−0.01	0.10	0.09 ***
Educational Board	0.06 **	−0.15 **	−0.03

COHRQoL: a child’s oral health-related quality of life, CPQ: Child Perception Questionnaire, SE: self-esteem, and CDA: child dental anxiety. * *p* < 0.05, ** *p* < 0.01, *** *p* < 0.001.

## Data Availability

Not applicable.

## Supplementary Materials

The following supporting information can be downloaded at: https://www.mdpi.com/article/10.3390/dj11070179/s1, Table S1: Correlation matrices for structural equation model (complete data for total sample).
