# Supplementary material for: The Associations among Dental Anxiety, Self-Esteem, and Oral Health-Related Quality of Life in Children: A Cross-Sectional Study
Source: Dent J (Basel). 2023 Jul 21;11(7):179. doi: 10.3390/dj11070179 (PMC10378345; doi:10.3390/dj11070179)
Supplement: Supplementary file 1 [file dentistry-11-00179-s001.zip › dentistry-2375123-supplementary.pdf]

Supplementary Materials

Table S1. Correlation matrices for structural equation model (complete data for total sample).

	**CPQ1**	**CPQ2**	**SE1**	**SE1**	**CDA1**	**CDA2**	**CDA3**	**Mean**	**SD**
CPQ1 ¥	1.00							16.67	7.03
CPQ2	0.59 ***	1.00						20.38	7.40
SE1 ∞	−0.28 ***	−0.20 ***	1.00					3.71	1.40
SE2	−0.14 ***	−0.06 *	0.08 ***	1.00				2.53	0.81
CDA1 ⁂	0.15 ***	0.21***	−0.05 *	0.01	1.00			19.37	8.12
CDA2	0.19 **	0.17 ***	−0.13 ***	−0.10 ***	0.69 ***	1.00		8.27	4.15
CDA3	0.09 ***	0.14 ***	−0.05 *	−0.06 *	0.50 ***	0.37 ***	1.00	5.45	2.68
Total sample = 1837									

¥ CPQ 1 and 2: Child Perception Questionnaire oral and functional factor (CPQ 1) and psychosocial (CPQ 2) subscales. ∞ SE 1 and 2: Self-esteem hesitant (SE 1) and confidence (SE 2) subscales ⁂ CDA 1, 2, 3: CFSS-DS dental fear (CD 1), hospital fear (CDA 2), and stranger fear (CDA 3) subscales * $p < 0.05$, ** $p < 0.01$, *** $p < 0.001$.
